# Making sense of SFX data: standards for data and structure validation for a non-standard experiment that has come of age

**DOI:** 10.1107/S2052252521006552

**Published:** 2021-06-30

**Authors:** Clyde A. Smith

**Affiliations:** a Stanford Synchrotron Radiation Lightsource, and Department of Chemistry, Stanford University, Menlo Park, CA, USA

**Keywords:** XFELs, serial femtosecond crystallography, data processing, validation

## Abstract

SFX diffraction data collection at XFELs is becoming more accessible. To extract the most useful biological information from these non-standard experiments, standards for SFX data analysis and structure validation must be redefined.

Innovation and revolution are paramount to the advancement of science and have shaped the ways in which we do research today. The field of structural biology has had multiple drivers of change, the two most recent being the use of X-ray free-electron laser (XFEL) sources for crystallographic data collection (Martin-Garcia *et al.*, 2016 ▸), and the ‘resolution revolution’ in cryo-EM, stemming from advances in detectors and image processing (Kühlbrandt, 2014 ▸). In protein crystallography, several advancements have moved this technique forward since the first structures of myoglobin and hemoglobin. These advances include (i) the use of synchrotron radiation (Dauter & Wlodawer, 2016 ▸; Phillips *et al.*, 1976 ▸); (ii) the application of multiwavelength data collection to the solution of the phase problem (Guss *et al.*, 1988 ▸; Hendrickson & Teeter, 1981 ▸; Hendrickson *et al.*, 1990 ▸); (iii) the advent of cryocrystallography (Hope, 1988 ▸; Pflugrath, 2015 ▸); (iv) automation (Cohen *et al.*, 2002 ▸; Snell *et al.*, 2004 ▸) and remote access (Smith *et al.*, 2010 ▸; Soltis *et al.*, 2008 ▸); and (v) the application of hybrid photon-counting (HPC) detectors (Förster *et al.*, 2019 ▸; Brönnimann & Trüb, 2018 ▸). Additional advances in data analysis and validation, including the use of the free *R* factor during refinement (Brünger, 1992 ▸), the introduction of *R*_meas_ and *R*_p.i.m._ statistics during data processing (Weiss, 2001 ▸) (which have effectively replaced *R*_merge_ in ‘Table 1’), and the use of CC_1/2_ and CC* (Karplus & Diederichs, 2012 ▸), have all contributed to the robustness of the modern protein crystallography experiment.

The most recent advance, the application of high-brilliance, time-structured XFEL beams to problems in structural biology, has disrupted the way in which the protein crystallography experiment is carried out at these fourth-generation light sources. The intense microfocus beams opened new experimental possibilities with micro- and nanocrystals, hitherto deemed too small for conventional data collection at synchrotrons, and the unique time-structure of the beams in the femtosecond regime, sparked a resurgence in the use of time-resolved (TR) crystallography to study the reaction mechanisms of enzymes in action (Tenboer *et al.*, 2014 ▸; Schmidt, 2017 ▸; Barends *et al.*, 2015 ▸). It also sparked the concomitant development of novel sample delivery methods including injectors, fixed target and hybrid methods (Martiel *et al.*, 2019 ▸). Moreover, the use of XFELs for protein crystallography has given rise to a new data collection paradigm, serial femtosecond crystallography (SFX), whereby a series of still images are collected from randomly oriented crystals intersecting the XFEL beam at a rate determined by the repetition rate of the beam and/or the readout rate of the detector.

Because the methods for data collection and data processing in conventional synchrotron crystallography were so robust, having been continuously developed over the preceding 50 or more years, it seemed obvious to attempt to apply these ‘standard’ methods to the data sets collected at XFELs. In this issue of **IUCrJ** , Gorel and colleagues (Gorel *et al.*, 2021 ▸) suggest that in SFX experiments the distinct features of the XFEL beams and the various ways in which samples are delivered into the beam give rise to issues unique to these types of experiments, particularly with respect to the determination of the quality of the data, the validity of the derived structure, and the extrapolated biological results. In order to fully validate the results from these experiments, the scientific community needs to be able to visualize and analyze the experimental data rather than relying on a ‘Table 1’ type of approach which, although completely adequate for conventional synchrotron-based diffraction experiments, falls short in the case of XFEL experiments.

A major challenge facing scientists using an XFEL, particularly those undertaking pump–probe TR studies in the picosecond and femtosecond regime, is the analysis of small structural changes of intermediates with low occupancy. The current study suggests that while some approaches to the analysis of TR experiments (for example, collecting alternating pump-ON and pump-OFF images and analyzing the data based on the ratio of *I*_ON_/*I*_OFF_) may work well for conventional TR studies, using an XFEL beam to reproduce this type of experiment generates non-systematic large differences which renders the *I*_ON_/*I*_OFF_ ratio method unusable. An alternative method looking at the intensity differences between an unpumped structure and various pumped structures at different timepoints can also fall short, particularly, as this study points out, since there seems to be some disagreement in the XFEL community as to the choice of the unpumped data set. In order to measure and have confidence in any biologically relevant structural changes stemming from a TR experiment, an estimate of the coordinate error in the structures at the various time points in a TR experiment is essential. Methods which rely on the refinement of multiple structures at each time point against resampled data sets are highlighted. These data sets are generated either by taking a subset of unique images (jackknifing) or by a random drawing with the replacement method, where the same images could be resampled for the same data set (bootstrapping) (Fig. 1 ▸).

We are now in a unique situation where we have on one hand a very well established set of protocols applicable to synchrotron-based crystallographic data collection, and on the other a novel approach to data collection at XFELs. Despite the limitations of the statistical conventions used to describe a traditional synchrotron data set when applied to an SFX data set, the two methods for diffraction data collection should not be seen as unrelated. Although SFX clearly has adopted some of the same methodologies employed at synchrotrons, particularly with respect to fixed target goniometer-based experiments (Cohen *et al.*, 2014 ▸), there has also been significant feedback from the XFELs, such that most synchrotron sources now have a microfocus serial synchrotron crystallography (SSX) capability (Pearson & Mehrabi, 2020 ▸). Since one of the main issues with XFELs is the scarcity and cost of beam time, it makes sense to use the relatively more abundant SSX beam time to measure serial data and undertake TR experiments from standard samples in order to establish protocols and obtain results which could be used to establish best practices and standards, as highlighted in this study. These could be used to drive software development and structure validation, which can then be applied to more complex ‘real world’ cases using SSX and SFX.

Finally, the authors point out that a large number of the structures generated from XFEL-based diffraction data are never fully refined and therefore not submitted to the PDB. It is suggested that the deposition of structure factors and map coefficients should become mandatory for structural papers which use data collected at XFELs, so that the scientific community could generate the same electron-density maps to validate the conclusions drawn by the researchers responsible for the data. Moreover, the raw data could be deposited to the Coherent X-ray Imaging Data Bank (CXIDB) so that the community could perform their own structure determinations (albeit a rather onerous task!) to confirm the results of any published study. These images could also be used in the development of data processing software and could aid in the establishment of best practices, protocols and reporting standards for this most non-standard of diffraction experiments.

## Figures and Tables

**Figure 1:**
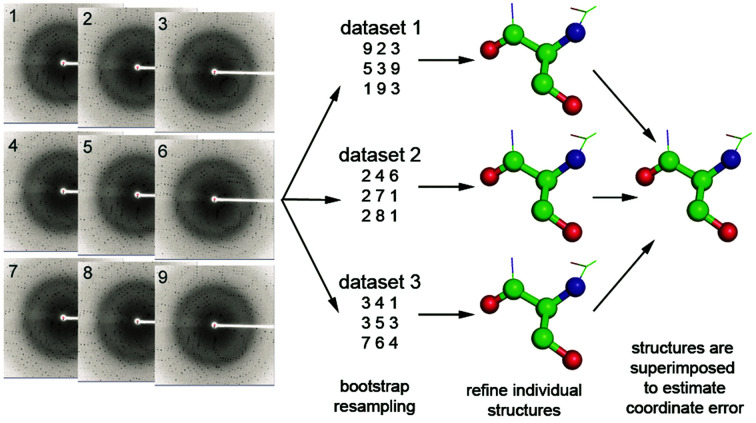
Bootstrap resampling (Grünbein *et al.*, 2021 ▸) by ‘random drawing with replacement’ is used to estimate the effect of measurement errors in SFX data on final refined coordinates. An image is randomly selected from the pool (left) and placed (as a copy) in ‘resampled’ data set 1. The image is then placed back in the original pool, and the pool is then randomly sampled again to add a second image to resampled data set 1. Multiple resampled data sets (up to 100 for example) are constructed that contain the same number of images as the original pool but in which images can be represented multiple times. Structures are determined from each of these resampled data sets, and then refined. The standard deviation of the ensemble-averaged bootstrapped structures gives the mean error of the coordinates. Although computationally time-consuming, bootstrapping can provide a valuable method of estimating coordinate error.
